# Supplementary material for: The Evolution and Characterization of the RNA Interference Pathways in Lophotrochozoa
Source: Genome Biol Evol. 2024 May 7;16(5):evae098. doi: 10.1093/gbe/evae098 (PMC11114477; doi:10.1093/gbe/evae098)
Supplement: evae098_Supplementary_Data [file evae098_supplementary_data.zip › Supplementary_materials.zip!/selected_miRNAs/NoGe_selected_miRNAs/mirdeep_pdf_files/NMRB01000201.1_Notospermus_geniculatus_strain_Ushimado_scaffold201,_whole_genome_shotgun_sequence_7102.pdf]

Provisional ID	:	NMRB01000201.1_Notospermus_geniculatus_strain_Ushimado_scaffold201,_whole_genome_shotgun_sequence_7102
Score total	:	1320.2
Score for star read(s)	:	3.9
Score for read counts	:	1312.4
Score for mfe	:	0.8
Score for randfold	:	0
Score for cons. seed	:	3
Total read count	:	2586
Mature read count	:	2492
Loop read count	:	0
Star read count	:	94

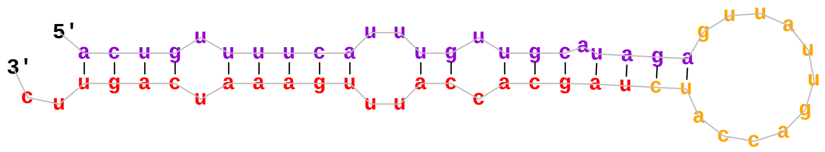

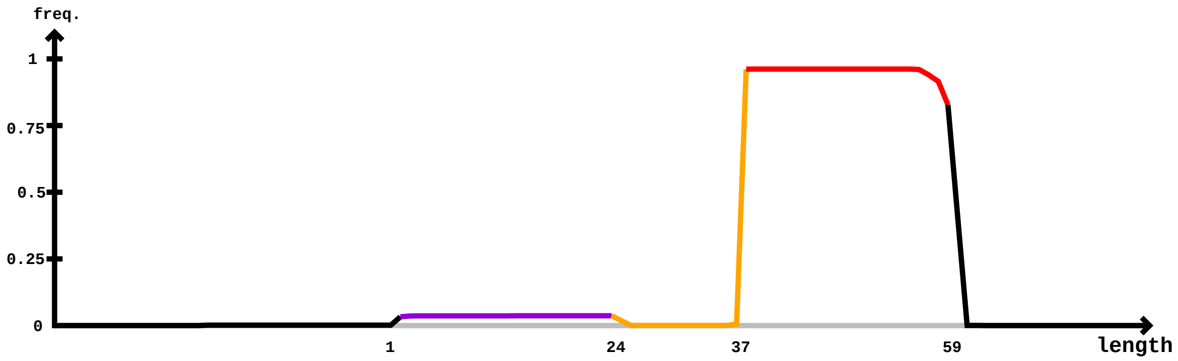


```
                                  Star                                  Mature
5'- ggagacuuucuugccgugauugccugucaaguguacuguuuucauuuguugcauagaguuauugaccaucuagcaccauuugaaaucaguucauuggagaggacaugcugcg  -3'   obs
    ggagacuuucuugccgugauugccugucaaguguacuguuuucauuuguugcauagaguuauugaccaucuagcaccauuugaaaucaguucauuggagaggacaugcugcg         exp
    .((((...))))((.((...(((((.((.((((.((((.(((((..((.(((.((((...........))))))).))..))))).)))).)))).)).))).)).)).)).   reads   mm   sample
    ..................................acuguuuucauuuguugcauaga.......................................................   29      0    Fm2
    ..................................acuguuuucauuAguugcauaga.......................................................   1       1    Fm2
    ...................................cuguuuucauuuguugcauaga.......................................................   6       0    Fm2
    ....................................................................cuGgcaccauuugaaaucaguu......................   12      1    Fm2
    ......................................................................uagcaccauuugaaaucaguu.....................   7       0    Fm2
    ......................................................................uagcaccauuugaaaucUguu.....................   1       1    Fm2
    ......................................................................uagcaccauuugaaauAaguuc....................   2       1    Fm2
    ......................................................................uagcGccauuugaaaucaguuc....................   1       1    Fm2
    ......................................................................uagcaccauuugaaaucCguuc....................   1       1    Fm2
    ......................................................................uagcaccauuAgaaaucaguuc....................   1       1    Fm2
    ......................................................................uagcaccauuugaaaucUguuc....................   1       1    Fm2
    ......................................................................uagcaccauuugaaaucaguuc....................   328     0    Fm2
    ......................................................................uUgcaccauuugaaaucaguuc....................   1       1    Fm2
    ......................................................................uagcaccauuugaaaucaguucU...................   9       1    Fm2
    ......................................................................uagcaccauuugaaaucaguGca...................   10      1    Fm2

    ..................................acuguuuucauuuguugcauaga.......................................................   10      0    Fm1
    ......................................................................uagcaccauuugaaauca........................   4       0    Fm1
    ......................................................................uagcaccauuugaaaucaguu.....................   3       0    Fm1
    ......................................................................uagcacUauuugaaaucaguuc....................   1       1    Fm1
    ......................................................................uagcaccauuugaaauUaguuc....................   1       1    Fm1
    ......................................................................uGgcaccauuugaaaucaguuc....................   3       1    Fm1
    ......................................................................uagcaccauuugaaaucaguuc....................   115     0    Fm1
    ......................................................................uagcaccauuugaaaucCguuc....................   1       1    Fm1
    ......................................................................uagcaccauuugaaaucGguuc....................   1       1    Fm1
    ......................................................................uagcaccauuugaaaucaguGca...................   3       1    Fm1
    ......................................................................uagcaccauuugaaaucaguucU...................   1       1    Fm1

    ......................................................................uagcaccauuugaaaucagu......................   3       0    Mm1
    ......................................................................uagcaccauuugaaauAaguu.....................   1       1    Mm1
    ......................................................................uagcaccauuugaaaucaguu.....................   81      0    Mm1
    ......................................................................uagcaccauuugaaaucCguu.....................   1       1    Mm1
    ......................................................................uGgcaccauuugaaaucaguuc....................   1       1    Mm1
```

```
                                  Star                                Mature
ggagacuuucuugccgugauugccugucaaguguacuguuuucauuuguugcauagaguuauugaccaucuagcaccauuugaaaucaguucauuggagaggacaugcugcg
......................................................................uagcaccauuugaaaucGguuc....................   1     1    Mm1
......................................................................uagcaccauuugaaaucUguuc....................   1     1    Mm1
......................................................................uagcaccauuugaaaucaguuc....................   138   0    Mm1
......................................................................uagcaccauuugaaaucaguuA....................   9     1    Mm1
......................................................................uagcaccauuugaaaucaguucU...................   3     1    Mm1
......................................................................uagcaccauuugaaaucaguuca...................   11    0    Mm1
......................................................................uagcaccauuugaaaucaguGca...................   10    1    Mm1

..................................acuguuuucauuuguugcauaga.......................................................   7     0    Mm2
....................................uguuuucauuuguugcauaga.......................................................   1     0    Mm2
......................................................................uagcaccauuugaaaucagu......................   1     0    Mm2
......................................................................uGgcaccauuugaaaucagu......................   3     1    Mm2
......................................................................uagcaccauuugaaaucaguu.....................   9     0    Mm2
......................................................................uagcaccauuAgaaaucaguuc....................   1     1    Mm2
......................................................................uagcaccauuugaaauGaguuc....................   1     1    Mm2
......................................................................uagcaccauuugaaaucCguuc....................   1     1    Mm2
......................................................................uagcaccauuugaaaucGguuc....................   2     1    Mm2
......................................................................uagcaccauuugaaaucaguuc....................   361   0    Mm2
......................................................................uagcaccauuGgaaaucaguuc....................   1     1    Mm2
......................................................................uagcaccauuugaaaucaguAc....................   1     1    Mm2
......................................................................uUgcaccauuugaaaucaguuc....................   1     1    Mm2
......................................................................uagcaccauuugaaaucaUuuc....................   1     1    Mm2
......................................................................uagcaccauuugaaauAaguuc....................   4     1    Mm2
......................................................................Nagcaccauuugaaaucaguuc....................   1     1    Mm2
......................................................................uagcaccauuugaaaucaguuU....................   1     1    Mm2
......................................................................uagcaccauuugGaaucaguuc....................   1     1    Mm2
......................................................................uagcaccauuugaaaucaguucU...................   17    1    Mm2
......................................................................uagcaccauuugaaaucaguGca...................   1     1    Mm2
......................................................................uagcaccauuugaaaucaguuca...................   5     0    Mm2

..............cgugauugccugucaagugu..............................................................................   4     0    Fm3
..................................acuguuuucauuuguugGauaga.......................................................   2     1    Fm3
..................................acuguuuucauuuguugcauaga.......................................................   28    0    Fm3
...................................cuguuuucauuuguugcauaga.......................................................   2     0    Fm3
.....................................................................cuagcaccauuugaaaucaguuc....................   1     0    Fm3
......................................................................uagcaccauuugaaaucag.......................   52    0    Fm3
......................................................................uagcaccauuugaaaucaU.......................   1     1    Fm3
......................................................................uGgcaccauuugaaaucagu......................   1     1    Fm3
......................................................................uagcaccauuugaaaucagu......................   27    0    Fm3
......................................................................uaUcaccauuugaaaucagu......................   4     1    Fm3
......................................................................uagcaccauuuAaaaucagu......................   1     1    Fm3
......................................................................uagcaccauuugaaaucaguA.....................   1     1    Fm3
......................................................................uagcaccauuugUaaucaguu.....................   1     1    Fm3
......................................................................uagcaccauuugaaaucaguu.....................   92    0    Fm3
......................................................................uagcaccauuugaaauAaguu.....................   1     1    Fm3
......................................................................uagcaccauuugaaaucGguu.....................   1     1    Fm3
......................................................................uagcaccauuugaaaucaguAc....................   1     1    Fm3
......................................................................uagcaccauuugaaauAaguuc....................   2     1    Fm3
......................................................................uagcaccauuGgaaaucaguuc....................   1     1    Fm3
......................................................................uagcaccauuugaaaucaguuc....................   438   0    Fm3
......................................................................uaAcaccauuugaaaucaguuc....................   2     1    Fm3
......................................................................uagcaccauuugaaauUaguuc....................   1     1    Fm3
......................................................................uagcaccauuugaaauGaguuc....................   1     1    Fm3
......................................................................uagcaccauuugaaaucaguuA....................   3     1    Fm3
......................................................................uagcaccauuugaaaucCguuc....................   1     1    Fm3
......................................................................uagcaccauuugaaaucaguucU...................   8     1    Fm3
......................................................................uagcaccauuugaaaucaguuca...................   4     0    Fm3
.......................................................................agcaccauuugaaaucaguuc....................   1     0    Fm3

..................................acuguuuucauuuguugGauaga.......................................................   1     1    Mm3
..................................acuguuuucauuuguugcauaga.......................................................   7     0    Mm3
..............................................uguugcauagaguuauuCaccauc..........................................   1     1    Mm3
......................................................................uGgcaccauuugaaaucagu......................   7     1    Mm3
......................................................................uagcaccauuugaaaucagu......................   16    0    Mm3
......................................................................uagcaccauuugaaaucaguu.....................   14    0    Mm3
......................................................................uagcaccauuugaaauAaguu.....................   1     1    Mm3
......................................................................uagcaccauuugaaaucaguuA....................   2     1    Mm3
......................................................................uagcaccUuuugaaaucaguuc....................   1     1    Mm3
......................................................................uagcaccauuugaUaucaguuc....................   1     1    Mm3
......................................................................uagcaccauuugaaaucaguuU....................   4     1    Mm3
```

```
                                 Star                                 Mature
ggagacuuucuugccgugauugccugucaaguguacuguuuucauuuguugcauagaguuauugaccaucuagcaccauuugaaaucaguucauuggagaggacaugcugcg
......................................................................uagcaccauuugaaaucCguuc....................    4     1     Mm3
......................................................................uagcaccauuugaaauAaguuc....................    4     1     Mm3
......................................................................uagcaccauuugaaaucaguuc....................    570   0     Mm3
......................................................................uagcaccauuGgaaaucaguuc....................    1     1     Mm3
......................................................................uagcaccauuugaaauGaguuc....................    1     1     Mm3
......................................................................Nagcaccauuugaaaucaguuc....................    1     1     Mm3
......................................................................uagcaccauuugaaaucaguuca...................    8     0     Mm3
......................................................................uagcaccauuugaaaucaguGca...................    2     1     Mm3
......................................................................uagcaccauuugaaaucaguucU...................    28    1     Mm3
......................................................................uagcaccauuugaaaucaguucUuu.................    1     1     Mm3
```
